# Elimination of an unfavorable allele conferring pod shattering in an elite soybean cultivar by CRISPR/Cas9

**DOI:** 10.1007/s42994-022-00071-8

**Published:** 2022-03-07

**Authors:** Zhihui Zhang, Jie Wang, Huaqin Kuang, Zhihong Hou, Pingping Gong, Mengyan Bai, Shaodong Zhou, Xiaolei Yao, Shikui Song, Long Yan, Yuefeng Guan

**Affiliations:** 1 College of Resources and Environment, Fujian Agriculture and Forestry University, Fuzhou, 350002 Fujian China; 2 FAFU-UCR Joint Center for Horticultural Biology and Metabolomics, Fujian Agriculture and Forestry University, Fuzhou, 350002 Fujian China; 3 College of Agriculture, Heilongjiang Bayi Agricultural University, Daqing, 163319 Heilongjiang China; 4 College of Life Sciences, Fujian Agriculture and Forestry University, Fuzhou, 350002 Fujian China; 5 The Key Laboratory of Crop Genetics and Breeding of Hebei, Institute of Cereal and Oil Crops, Hebei Academy of Agricultural and Forestry Sciences, Shijiazhuang, 050035 China

**Keywords:** Genome editing, Pod shattering, Soybean, Precision breeding, Marker assisted selection, CRISPR/Cas9

## Abstract

Pod shattering can lead to devastating yield loss of soybean and has been a negatively selected trait in soybean domestication and breeding. Nevertheless, a significant portion of soybean cultivars are still pod shattering-susceptible, limiting their regional and climatic adaptabilities. Here we performed genetic diagnosis on the shattering-susceptible trait of a national registered cultivar, Huachun6 (HC6), and found that HC6 carries the susceptible genotype of a candidate *Pod dehiscence 1* (*PDH1*) gene, which exists in a significant portion of soybean cultivars. We next performed genome editing on *PDH1* gene by clustered regularly interspaced short palindromic repeats (CRISPR)-CRISPR-associated protein 9 (Cas9). In T_2_ progenies, several transgene-free lines with *pdh1* mutations were characterized without affecting major agronomic traits. The *pdh1* mutation significantly improved the pod shattering resistance which is associated with aberrant lignin distribution in inner sclerenchyma. Our work demonstrated that precision breeding by genome editing on *PDH1* holds great potential for precisely improving pod shattering resistance and adaptability of soybean cultivars.

Dear Editor,

Conventional breeding has been playing a fundamental role in crop improvement in the past century. Nevertheless, incorporating beneficial genetic variations while excluding unfavorable alleles remains a major challenge, impacted by recombination rates, population size, allelic variations, and effectiveness of phenotypic selection (Lyzenga et al. 2021). As a result, an elite cultivar may take 5–10 years to develop, yet still carry substantial unfavorable allelic variations. Genome editing technologies, particularly clustered regularly interspaced short palindromic repeats (CRISPR)/associated (Cas) nucleases (CRISPR/Cas), can facilitate knock-out, knock-in, and base-editing of target genes, opening up new possibilities to precise improvement of crops (Cai et al. 2020; Chen et al. 2019; Li et al. 2020; Tang et al. 2019; Wang et al. 2020b). The development of CRISPR/Cas-based gene editing has created an avenue for creation of favorable alleles or elimination of maladapted genetic variations in germplasm, before or after the breeding cycle (Lyzenga et al. 2021).

Pod shattering has been an unfavorable trait in soybean (*Glycine max*) breeding, yet is inapparent in humid climate. As a result, a significant portion of soybean cultivars and landraces are still pod shattering-susceptible in areas with higher humidity (Zhang and Singh 2020). The adaptability of shattering-susceptible varieties is severely limited by local climates and is not suitable for introduction to arid regions. For instance, Huachun 6 (HC6) is a national registered cultivar in south China featuring good yield performance and high protein content, yet is shattering-susceptible. The manual harvesting practice and high humidity in south China helped avoid shattering, so yield performance of HC6 is not significantly affected (Fig. 1A). However, in HuangHuaiHai (HHH) region where HC6 can be adapted as summer sowing variety, the low humidity and machine harvest can cause severe yield losses of HC6 (Fig. 1B), limiting its regional adaptability.

**Fig. 1:**
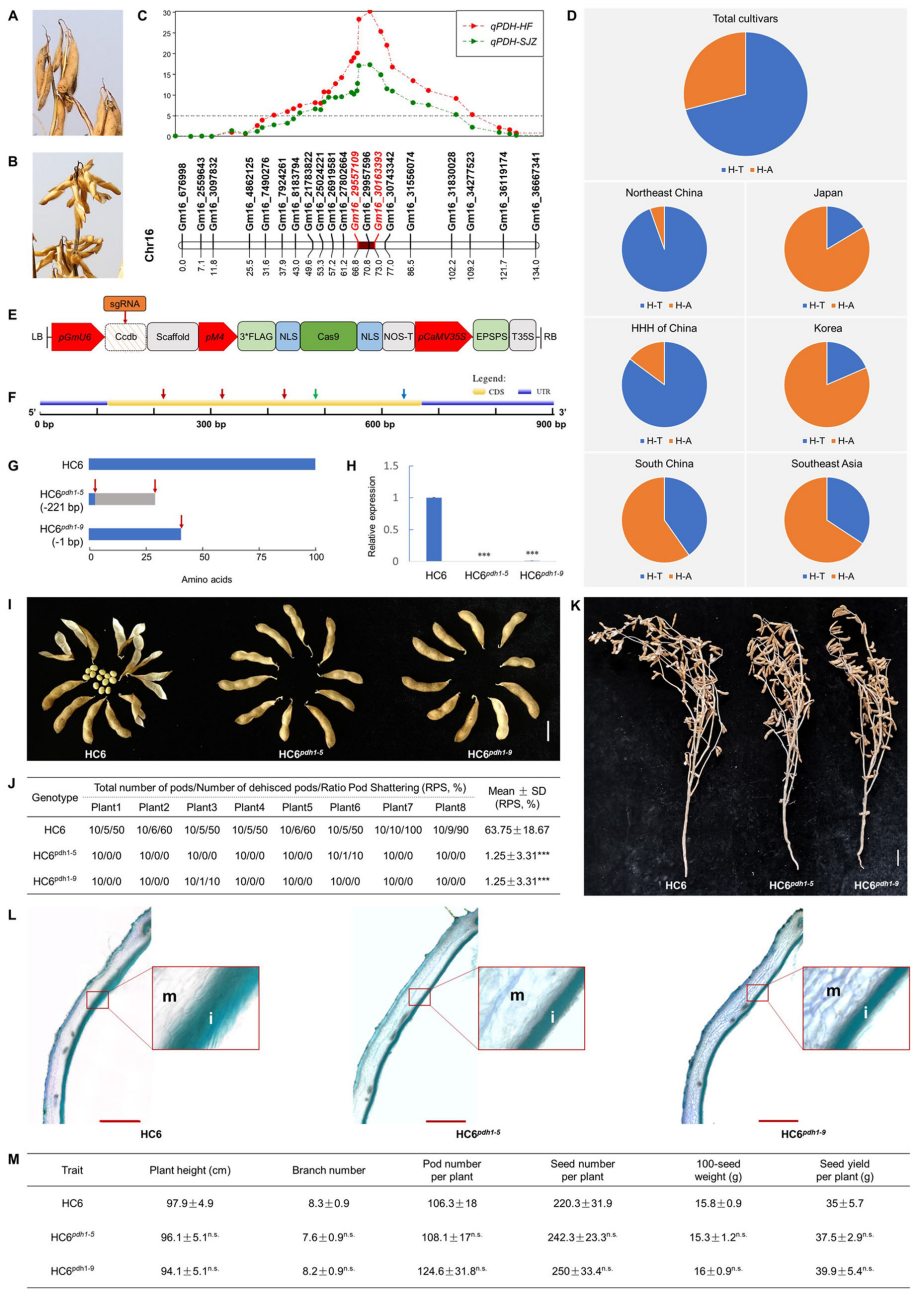
Genetic diagnosis and elimination of *PDH1* in HC6. **A**, **B** The field performance of HC6 in **A** Fujian, China (E 119.24°, N 26.08°) and **B** Hebei, China (E 114.78°, N 37.91°). **C** QTL identification for pod shattering resistance. QTLs are represented by bars (1-LOD interval) and extended lines (2-LOD interval). The dotted line represented the threshold value. **D** Allelic frequency of the nonsense SNP A-T of *PDH1*, in China, Japan, Korea, and southeast Asia. A total of 1080 soybean cultivars were analyzed from various origins, including 346 from northeast China, 418 from HuangHuaiHai (HHH) region of China, 72 from south China, 55 from Japan, 119 from Korea, 70 from southeast Asia. **E** The architecture of pGES701 vector. **F** Gene structure, sgRNA target sites (represented by red arrow), and the positions of primers (represented by green arrow (forward primer) and blue arrow (reverse primer)) used in qRT-PCR in *PDH1*. **G** Predicted protein structures of HC6 and mutants (HC6^*pdh1−5*^ and HC6^*pdh1−9*^). Red arrow represents the position of frameshift or premature stop mutation. **H** Relative expression of *PDH1* in pod walls of HC6 and mutants. Asterisks indicate significant differences (*P*< 0.001, by Student’s *t* test). **I** Dried pods of HC6 and mutants kept in a circulation drier at 60 °C for 6 h. Scale bar = 2 cm. **J** Percentages of dehisced pods of HC6 and mutants after keeping in a circulation drier at 60 °C for 6 h. **K** The field performance of HC6 and mutants in Shijiazhuang, Hebei, China (E 114.78°, N 37.91°). Scale bar = 5 cm. **L** Cross-section of matured pod wall of HC6 and mutants. *m* mesocarp; *i* inner sclerenchyma. Scale bar = 1 mm. **M** The agronomic traits of HC6 and mutants in the field. Values are shown in mean ± SD (*n* = 7–10). *n.s.* not significant (*P*> 0.05 by Student’s *t* test)

To diagnose the genetic basis of pod shattering susceptibility in HC6, we performed QTL mapping with a recombinant inbred line (RIL) population of HC6 and pod shattering-resistant JD12. A reproducible major QTL controlling shattering resistance was mapped to chromosome 16, which overlapped with the previously reported *qPDH1* QTL (Fig. 1C). The putative *PDH1* gene was proposed as *Glyma16g25580* (Wm82.a1.v1) encoding a dirigent (DIR) family protein expressed in the inner sclerenchyma of pod walls in shattering susceptible varieties (Funatsuki et al. 2014). We sequenced *Glyma16g25580* and found a SNP in JD12 (chr16-29944393, A/T (HC6/JD12); Wm82.a2.v1) leading to a nonsense variant, consistent with a previous report that *Glyma16g25580* exists as a truncated gene in shattering-resistant cultivars (Funatsuki et al. 2014). According to the nonsense SNPs A-T, we surveyed the haplotypes of *PDH1* gene among resequencing data from 1080 soybean cultivars. The proposed shattering-resistant H-T haplotype is largely fixed in regions with low relative humidity and mechanic harvesting, including 94.50% in northeast China and 85.17% in HHH region (Fig. 1D). In contrast, the shattering-susceptible H-A haplotype is retained in areas with relatively high humidity and/or manual harvesting, including 59.72% of south China, 83.64% of Japan, 81.51% of Korea, and 65.71% of southeast Asia cultivars (Fig. 1D). This result suggested that the presence of *Glyma16g25580* gene is highly associated with the relative humidity and harvesting mode shaped pod shattering trait.

Genome editing technologies, particularly clustered regularly interspaced short palindromic repeats (CRISPR)/associated (Cas) nucleases (CRISPR/Cas), can facilitate knock-out, knock-in, and base-editing of target genes, creating an avenue for elimination of maladapted genetic variations in germplasm (Cai et al. 2020; Chen et al. 2019; Li et al. 2020; Tang et al. 2019; Wang et al. 2020b). The *Glyma16g25580* (designated *PDH1* thereafter) open reading frame is mainly responsible for the pod shattering susceptible trait. We then sought to generate mutation of *PDH1* by CRISPR/Cas9 in HC6. We designed three sgRNAs, cloned into pGES701 vector individually, and pooled for Agrobacterium-mediated transformation (Fig. 1E–F). Among 23 T_0_ transgenic plants, 17 lines contained sgRNA, 5 lines contained 2 sgRNAs, and 1 contained all sgRNAs. Hi-TOM (Liu et al. 2019) and sanger sequencing analysis showed that 10 of the 23 T_0_ transgenic plants carried mutations in at least one target locus. In T_1_ progenies, we characterized homozygous mutant plants from two lines, HC6^*pdh1−5*^ with a 221 bp deletion and HC6^*pdh1−9*^ with 1 bp deletion, respectively (Fig. 1G). qRT-PCR showed that the expression of *PDH1* gene was diminished by homozygous mutations in both lines (Fig. 1H). In T_2_ progenies, homozygous mutant plants without transgene were characterized in both lines (data not shown).

In a heat dried assay, HC6 pods displayed a high ratio of shattering (Ratio Pod Shattering = 63.75%, *n* = 8; Fig. 1I–J). In contrast, HC6^*pdh1−5*^ and HC6^*pdh1−9*^exhibited significant resistance to pod shattering (RPS = 1.25%, *n* = 8; Fig. 1I–J). The dehisced pod walls of HC6^*pdh1−5*^ and HC6^*pdh1−9*^ exhibited much lower degrees of torsion than those of HC6, which is consistent with previous finding that *qPDH1* locus was associated with curling of pod walls (Funatsuki et al. 2014). We then analyzed the anatomical characteristics of HC6^*pdh1−5*^, HC6^*pdh1−9*^and HC6 pods. We found that the lignin layer in inner sclerenchyma of HC6 tends to be thicker and looser. In contrast, the lignin layer in HC6^*pdh1−5*^ and HC6^*pdh1−9*^ appeared to be thinner and compact (Fig. 1L). This result demonstrated that genome editing of the *PDH1* gene may affect the pod shattering resistance of HC6 by influencing the deposition of lignin layer in inner sclerenchyma. In 2021 summer, we performed a field trail at Shijiazhuang, Heibei, China. When harvesting was delayed for 2 weeks, HC6 exhibited significant pod shattering that caused substantial yield losses. In contrast, HC6^*pdh1−5*^ and HC6^*pdh1−9*^ barely showed shattering at the same condition (Fig. 1K). Meanwhile the genome editing of PDH1 did not significantly affect other agronomic traits, including plant height, branch number, pod number per plant, seed number per plant, 100-seed weight, and seed yield per plant (as determined before pod shattering occurs in HC6) (Fig. 1M).

Here we showcased the genetic diagnosis and “gene therapy” of the pod shattering trait of soybean by CRISPR/Cas9 genome editing. In comparison with introgression breeding, genome editing approach could rapidly and precisely improve a trait, and is not limited by genetic diversity of breeding populations (Chen et al. 2019; Li et al. 2020; Manghwar et al. 2019). Therefore, genome editing can be integrated as a routine part of a breeding cycle to eliminate unfavorable alleles (such as *PDH1*) to facilitate the generation of a genetically superior cultivar.

## Materials and methods

### Genomic editing of soybean *PDH1* by CRISPR/Cas9

CRISPR/Cas9 mutations of *PDH1* in HC6 was performed using the protocol published previously (Bai et al. 2020), and a new CRISPR/Cas9 vector, pGES701 (Fig. 1E), was used for genome editing. The mixed *Agrobacterium* solution was transformed into the soybean cultivar HC6 via *A. tumefaciens*-mediated transformation, as described previously (Bai et al. 2020).

### RNA extraction and real-time qPCR

To measure the expression of *PDH1* gene in WT plants and mutants, real-time qPCR was performed using total RNA extracted from pod wall samples (3 weeks after flowering). Total RNA extraction, cDNA synthesis, and data analysis were performed as previously described (Wang et al. 2020a).

### Evaluation of pod dehiscence percentage

The pod dehiscence percentage of WT and mutations was evaluated by heat treatment: ten fully matured pods of each plant were collected and kept in a circulation drier at 60 °C for 6 h and then counted the number of dehisced pods, respectively. Eight plants per genotype were sampled. Fully matured pods of WT and mutations were examined for pod-wall lignification. Soybean pod was embedded in 7% agarose and cross-sections (80 μm thick) were stained with 10% toluidine blue, and observed under a microscope (Eclipse Ni-U, Nikon, Japan).
